# Evolution of Retinal Morphology Changes in Amyotrophic Lateral Sclerosis

**DOI:** 10.3390/jcm15010258

**Published:** 2025-12-29

**Authors:** Valeria Koska, Stefanie Teufel, Aykut Aytulun, Margit Weise, Marius Ringelstein, Rainer Guthoff, Sven G. Meuth, Philipp Albrecht

**Affiliations:** 1 Department of Neurology, Maria-Hilf-Clinics, 41063 Mönchengladbach, Germany; 2 Department of Anesthesiology and Intensive and Palliative Care, Städtisches Klinikum Solingen, 42653 Solingen, Germany; 3 Department of Psychiatry, LVR-Klinikum, Heinrich-Heine-University Düsseldorf, 40204 Düsseldorf, Germany; 4 Department of Neurology, Medical Faculty, Heinrich-Heine-University Düsseldorf, 40225 Düsseldorf, Germany; 5 Department of Neurology, Center for Neurology and Neuropsychiatry, LVR-Klinikum, Heinrich-Heine-University Düsseldorf, 40629 Düsseldorf, Germany; 6 Department of Ophthalmology, Medical Faculty, University Clinic of Heinrich Heine University Düsseldorf, 40204 Düsseldorf, Germany; rainer.guthoff@med.uni-duesseldorf.de

**Keywords:** neurodegeneration, optic coherence tomography, retina

## Abstract

**Background/Objectives**: To compare changes in the thickness of retinal layers between patients with amyotrophic lateral sclerosis (ALS) and healthy controls using optical coherence tomography. Amyotrophic lateral sclerosis is a degenerative disease of the upper and lower motoneurons with a rapidly progressive course, but non-motor symptoms such as decreased ocular motility and reduced visual acuity have also been reported. Specific biomarkers or surrogate parameters assessing neurodegeneration in ALS are of interest. **Methods**: In a retrospective, longitudinal study using optic coherence tomography of the retinal layers, we compared changes in the thickness of the layers between patients with ALS and healthy controls. Correlations to clinical scores, such as the modified ranking scale, were analyzed. **Results**: In our cohort of patients with early ALS (disease duration 5.15 ± 21.4 months at baseline), we neither observed differences in retinal layer thickness at baseline nor did the thickness changes in any retinal layer differ in comparison to healthy controls at baseline. Moreover, we observed no significant thickness changes over the course of the observational period in our patients with ALS. However, a correlation analysis revealed a negative association of the thickness change rates in the complex of ganglion cell and inner plexiform layer and the inner nuclear layer with a higher modified Rankin scale at follow-up. **Conclusions**: This study adds to the notion that OCT may not be a suitable tool to monitor atrophy and disease progression in ALS. However, further longitudinal studies with longer follow-up times and larger cohorts are warranted.

## 1. Introduction

Amyotrophic lateral sclerosis (ALS) is a degenerative disease of the upper and lower motoneurons with a rapidly progressive course often leading to death within two to five years [1]. It combines symptoms of an upper motoneuron loss, such as spasticity and a positive Babinski’s reflex, and symptoms of a lower motoneuron damage, including limb paresis and muscle atrophy [2]. Most patients also show signs of bulbar involvement, such as dysarthria and dysphagia [3]. Increasing evidence from clinical and pathological studies suggests a variety of non-motor symptoms as frontotemporal dementia, visual impairment [4], or fatigue [5]. Recently, a new algorithm for diagnosis of ALS has been developed [6]. The clinical status of patients with ALS can be assessed using different scores: the modified Rankin scale (mRS) describes mental and physical limitations in a seven-step scale. It was developed to describe the outcome of stroke patients and can also be used to describe patients with ALS [7]. The Barthel index (BI) describes limitations in activities of daily life [8]. Disease-specific limitations can be assessed using the ALS-Functional Rating Scale (ALS-FRS) [9], with lower points describing a more severe handicap. The ALS-FRS is also used as a progression parameter over the course of the disease in clinical trials [10,11,12]. Negative prognostic markers are a bulbar onset, weight loss, executive dysfunction, and older age at onset [13,14]. Specific biomarkers or surrogate parameters assessing neurodegeneration in ALS are still lacking. Optical coherence tomography (OCT) detected retinal thickness changes in multiple sclerosis [15] and neurodegenerative disorders such as Alzheimer’s [16] and Parkinson’s [17] disease. In a previous cross-sectional study, we described a reduced thickness of the macular retinal nerve fiber layer (mRNFL), inner nuclear layer (INL), and total macular thickness (TMT) in patients with ALS [18]. In this longitudinal study, we aimed to investigate the relationship between retinal layer thickness changes using OCT imaging and the change in the clinical status of patients with ALS. Possible relations might be used as a biomarker for clinical studies in the future.

## 2. Materials and Methods

In this retrospective longitudinal study, possible changes in the retinal layers were analyzed by comparing OCT datasets of patients diagnosed with ALS with healthy controls (HC). All subjects gave their written and informed consent. Patients with retinopathy, glaucoma, or myopia or hyperopia of more than 5 diopters were excluded from the study. Further exclusion criteria were other neurological, inflammatory, or degenerative diseases in the medical history. Healthy controls were included in the study site, applying the same exclusion criteria. For all patients, clinical scores including the mRS, BI, and ALS-FRS were obtained at baseline and follow-up, if possible.

**Figure 1:**
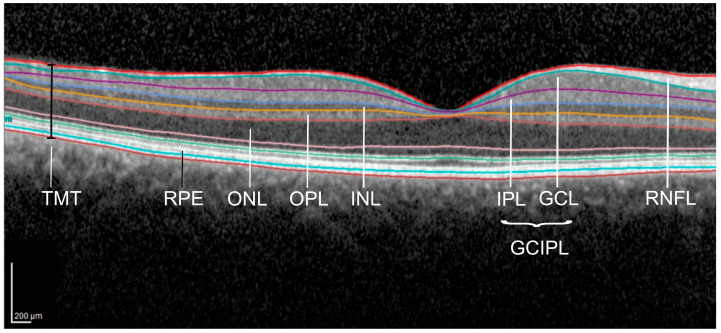
Horizontal transfoveal optic coherence tomography scan depicting the macular retinal layers. Scans were analyzed using the automatic segmentation by Heidelberg Eye ExplorerTM, and errors were corrected manually. TMT = total macular thickness; RNFL = retinal nerve fiber layer; GCIPL = complex of GCL = ganglion cell layer + IPL = inner plexiform layer; INL = inner nuclear layer; OPL = outer plexiform layer; ONL = outer nuclear layer; RPE = retinal pigment epithelium.

We report the OCT methodology according to the APOSTEL recommendations [19,20]. All scans were obtained using a SpectralisTM OCT (Heidelberg Engineering, Heidelberg, Germany) device under ambient light conditions without dilation of the pupil. Using vertical high-resolution volume scans after centering in the middle of the fovea, the total macular thickness and the mRNFL thickness could be detected using the segmentation algorithm of the OCT device. In a second high-resolution volume scan, the volume of the inner and outer retinal layers (complex of ganglion cell and inner plexiform layer = GCIPL; inner nuclear layer = INL; outer plexiform layer = OPL; outer nuclear layer = ONL; retinal pigment epithelium = RPE) was detected. Macular volume scans were analyzed using the automatic segmentation by Heidelberg Eye ExplorerTM (Software version V6.16.2), and obvious errors were corrected manually afterwards (see Figure 1). Using the 1, 3, and 6 mm Early Treatment Diabetic Retinopathy Study (EDTRS) grid, the mean macular thickness was calculated for each layer. The peripapillary RNFL (pRNFL) thickness was obtained using a peripapillary ring scan with a diameter of 3.5 mm (100 A-scans). The examiner positioned the circle around the optic nerve head manually. All scans fulfilled the OSCAR-IB-Criteria [21].

Statistical analysis was performed using Microsoft Excel and SPSS Statistics (26.0) or GraphPad Prism (8.00). Primary outcome parameters were differences in baseline thickness and annualized changes in the pRNFL and the macular retinal layers. OCT data were obtained at baseline and on the follow-up visit. Baseline demographic data were compared between patients and healthy controls, and age was tested using a two-tailed t-test and sex using a chi-squared test. Baseline retinal thicknesses were compared between the patients with ALS and healthy controls using a generalized estimation equation (GEE) model with an exchangeable intercorrelation matrix accounting for within-subject inter-eye correlations and correcting for sex. Annualized thickness change rates were calculated from longitudinal data by linear regression analysis, and group comparison was performed using the Mann–Whitney U test. Using the same GEE model as before, follow-up thickness was compared to baseline for each layer for patients with ALS. Because of small numbers in HCs, longitudinal analysis was performed using the Wilcoxon test, comparing the mean of both eyes. A subanalysis controlling for disease duration in patients with ALS was added. A possible change in the clinical scores (mRS, BI, and ALS-FRS) between baseline and follow-up visit was analyzed using the Wilcoxon test. Using Spearman’s Rho correlations, the relation between retinal thickness layers and the clinical scores (mRS, BI, and ALS-FRS) at baseline was analyzed. The same analysis was performed for the relationship between annual retinal thickness change rates and the clinical scores at follow-up.

## 3. Results

### 3.1. Demographics

In this retrospective longitudinal study, we identified 22 patients diagnosed with ALS and 21 healthy controls (HCs), who were analyzed at baseline. All patients with ALS and 5 of the HCs received longitudinal OCT measurements. The baseline and follow-up visits were 5.4 ± 0.5 months (mean ± SEM) apart for patients with ALS (median 5.0 months) and 87.4 ± 4.8 months for healthy controls (median 91 months) (U = 0.0; Z = −4.943; *p* < 0.001, Mann–Whitney U test). The age of patients with ALS and HCs did not differ (63 ± 2 vs. 60 ± 1 years, U = 712, z = −1.835, *p* = 0.067, Mann–Whitney U test; age was not normally distributed in patients with ALS: patients with ALS *p* < 0.001, HCs *p* = 0.075). Sex distribution was reversed between both groups, with 18% female patients with ALS and 48% female HCs (*p* = 0.004, chi-square test). The GEE model was, therefore, adjusted for sex. Disease duration at baseline was obtained for 20 patients with ALS, with a mean of 5.15 ± 21.4 months. We did not have any information on genetic testing of the participants.

### 3.2. OCT Data

**Figure 2:**
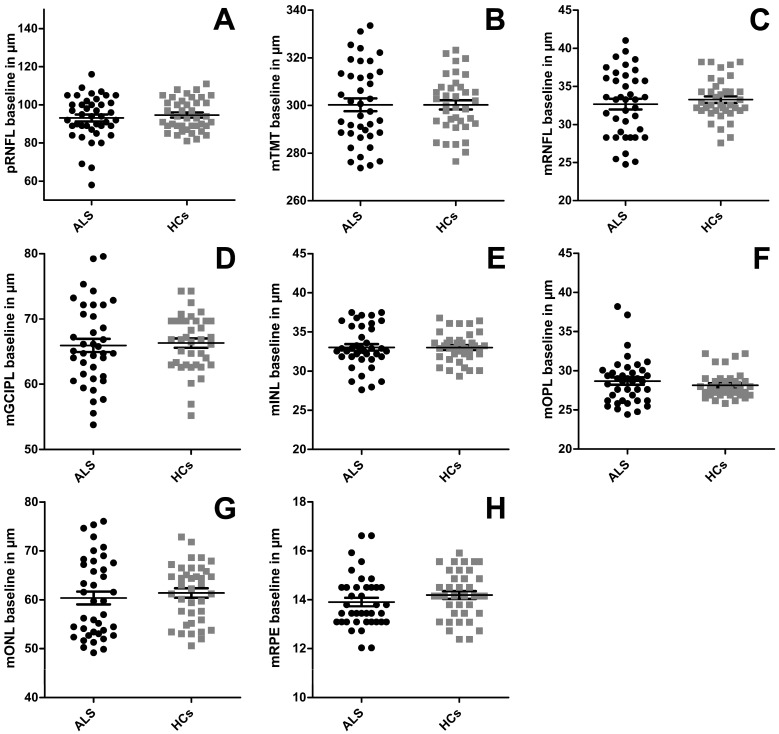
OCT results at baseline. The baseline thicknesses for the retinal layers are displayed for patients with ALS and healthy controls (HC). (**A**) pRNFL = peripapillary retinal nerve fiber layer; (**B**) TMT = total macular thickness; (**C**) mRNFL = macular retinal nerve fiber layer; (**D**) mGCIPL = complex of GCL = ganglion cell layer + IPL = inner plexiform layer; (**E**) mINL = inner nuclear layer; (**F**) mOPL = outer plexiform layer; (**G**) mONL = outer nuclear layer; (**H**) mRPE = retinal pigment epithelium. Each dot represents one eye. Mean and standard error of the mean (SEM) are depicted with the lines and antennae. Group comparison was conducted using a GEE model accounting for within-subject inter-eye correlations and correcting for sex. There were no significant differences between the two groups.

**Figure 3:**
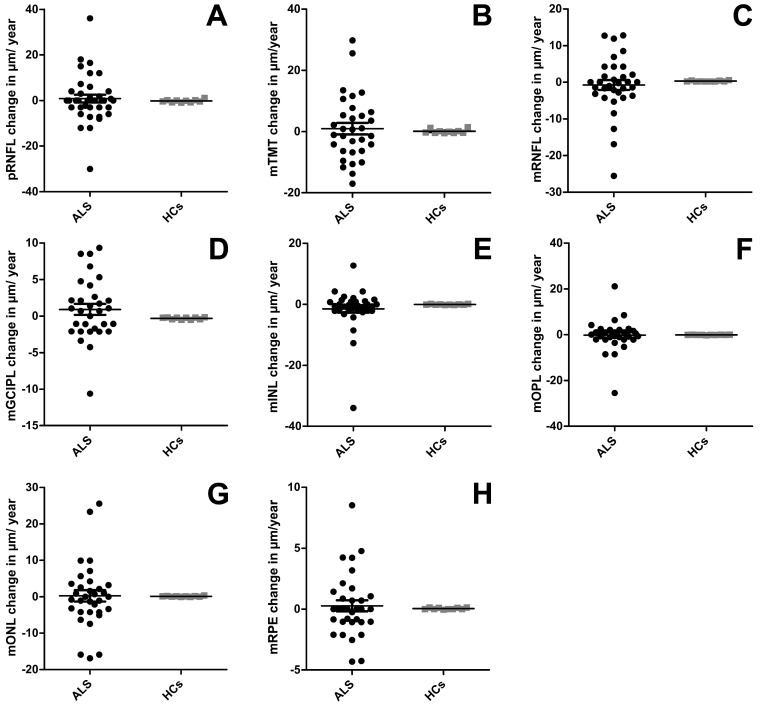
OCT results at follow-up. The annualized change rates after 12 months are displayed for each layer for patients with ALS and healthy controls (HC). (**A**) pRNFL = peripapillary retinal nerve fiber layer; (**B**) TMT = total macular thickness; (**C**) mRNFL = macular retinal nerve fiber layer; (**D**) mGCIPL = complex of GCL = ganglion cell layer + IPL = inner plexiform layer; (**E**) mINL = inner nuclear layer; (**F**) mOPL = outer plexiform layer; (**G**) mONL = outer nuclear layer; (**H**) mRPE = retinal pigment epithelium. Each dot represents one eye. Mean and standard error of the mean (SEM) are depicted with the lines and antennae. Group comparison was conducted using the Mann–Whitney U test. There were no significant differences between the two groups.

We observed no significant differences between the patients with ALS and HC at baseline. All layers apart from the OPL were slightly thinner in patients with ALS (see Table 1 and Figure 2), but without reaching significance. A subanalysis including disease duration at baseline into the GEE model did not change the results of the main analysis; the disease duration had no significant influence on any parameter. The longitudinal analysis did not reveal a significant thinning between baseline and follow-up visit in patients with ALS (see Table 1) when using the GEE model. After calculating the annual change rate of all retinal layers, the group comparison using the Mann–Whitney U-test showed no difference between patients with ALS and HCs (see Table 1 and Figure 3).

### 3.3. Clinical Scores

The mRS, which describes the physical and mental limitations of the patients, was obtained for all patients at both visits, with a median of 2 points at baseline and 2.5 points at follow-up. We observed a significant deterioration in the mRS between both visits (z = −3.051, *p* < 0.001, Wilcoxon). The BI was available for 9 patients at baseline (median 100 points) and for 11 patients at follow-up (median 95 points) with no significant difference between both visits (z = −1.342, *p* = 0.180, Wilcoxon). The ALS-FRS was available for six patients at baseline (median 29 points) and eight patients at follow-up (median 27 points) without a significant change (z = −1.841, *p* = 0.066, Wilcoxon).

**Figure 4:**
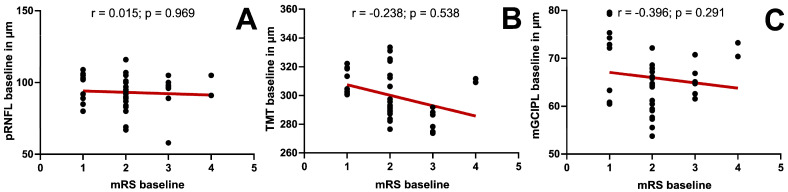
Correlation between retinal layer thickness and clinical scores at baseline. Using Spearman’s Rho, a correlation between baseline retinal layer thickness and clinical scores was examined. (**A**) Modified Rankin scale (mRS) and peripapillary retinal nerve fiber layer (pRNFL). (**B**) mRS and total macular thickness (TMT). (**C**) mRS and complex of macular ganglion cell inner plexiform layer (mGCIPL). The results were considered significant with *p* ≤ 0.05.

We observed no significant correlations between any of the clinical scores and the inner and/or outer macular retinal layers and the pRNFL (see Figure 4).

**Figure 5:**
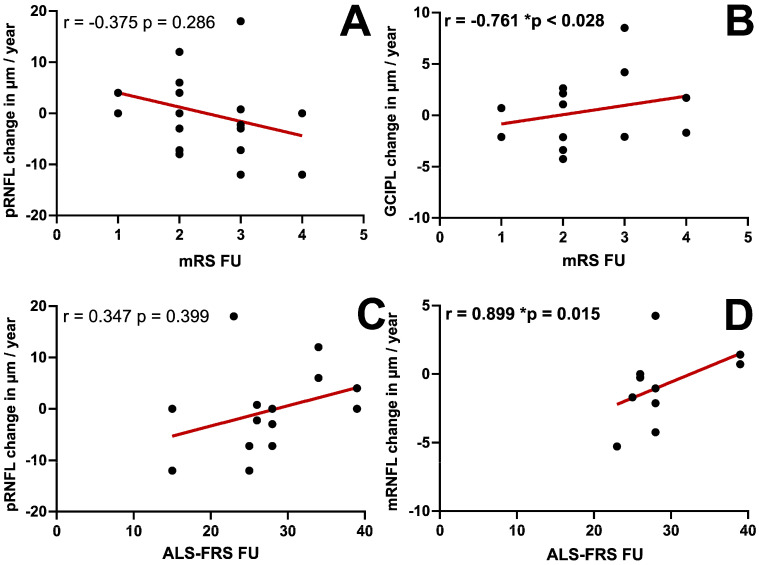
Correlation between retinal layer thickness and clinical scores at follow-up. Using Spearman’s Rho, a correlation between the annualized retinal layer thickness change after 12 months and the clinical scores at follow-up (FU) was examined. (**A**) Modified Rankin scale (mRS) and peripapillary retinal nerve fiber layer (pRNFL). (**B**) mRS and complex of macular ganglion cell and inner plexiform layer (GCIPL). (**C**) ALS functional rating scale (ALS-FRS) and pRNFL. (**D**) ALS-FRS and macular retinal nerve fiber layer (mRNFL). The results were considered significant with * *p* ≤ 0.05.

After conducting a similar analysis, including the annual change rates of all retinal layers and the clinical scores at the follow-up visit, the mRS showed a significant negative correlation with the annual change rate of the GCIPL (r = <0.761; *p* = 0.028, Figure 5B). There was no correlation between the BI and any of the annual change rates (Table 2). The ALS-RFS correlated significantly with the annual change rate of the mRNFL (r = 0.899; *p* = 0.015, Figure 5D) (see Table 2 for detailed information).

## 4. Discussion

Even though other studies have shown differences in change rates of retinal layer thickness compared to controls measured by OCT, our study could not detect longitudinal thickness changes in patients with ALS. Our baseline data is in line with some previous studies. A large cross-sectional study found neither any difference in macular layer thicknesses nor a correlation to disease severity [22]. Rojas et al. described a significantly increased TMT only in the inferior and temporal inner macular ring segment of the EDTRS grid at baseline in patients with ALS compared to HCs, while the cube average thickness of the macula, like in our study, showed no difference (*p* = 1.00) [23]. At the follow-up visit after six months in that study, TMT was significantly thinner only for the inner and the outer inferior macular ring segment in the patients with ALS when compared to baseline [23]. Rohani et al. examined the peripapillary RNFL and found that the average pRNFL thickness was significantly lower in patients with ALS compared to controls [24]. Contrasting our current findings, in a previous cross-sectional study, we observed a subtly but significantly thinner macular and peripapillary RNFL and the INL in patients with ALS compared to HCs [18]. A metanalysis performed by Nepal et al. [25] also reported no differences in retinal nerve fiber layer thickness, but a significantly thinner inner nuclear layer thickness in patients with ALS. The reasons for the difference in cross-sectional data between our study from 2014 and the current investigation may be explained by the shorter disease duration in our study (5.15 ± 21.4 months vs. 22.3 ± 22.57 months in 2014). Considering all studies, we believe that differences in retinal morphology between patients with ALS and age-matched HCs are likely to be very small or negligible. However, a histological examination of patients with ALS and transgenic ALS/dementia mice demonstrated intraretinal deposits and loss of ganglion cell axons, respectively [26], which might explain a retinal thickness increase at some point in the clinical course of ALS. Their OCT analysis described a thinner TMT and peripapillary RNFL in comparison to HCs and an inverse correlation with disease duration for both parameters. Mean disease duration was longer compared to our data set, with a mean of 42 ± 34 months, but a range of 7 to 166 months, while Roth et al. (10.80 ± 5.5 months) and we (5.15 ± 21.4 months) included patients in an earlier stage of the disease. Retinal thickness changes might mainly occur in the later course of the disease and might not appear in longitudinal analyses with short intervals or early in the disease. 

In this study, we observed no significant correlation between the clinical scores and baseline retinal layer thicknesses. The mRS was the only clinical score assessed for all patients at both visits, thus enabling a reliable correlation analysis. This longitudinal analysis revealed a negative correlation between the mRS and GCIPL thickness change. Correlation analyses for the BI and ALS-RFS should not lead to any interpretation, as they were applicable to only very few of our patients. Rojas et al., on the other hand, reported a reverse correlation between the peripapillary RNFL and the ALS-FRS, with a lower RNFL being associated with a higher score in the ALS-FRS and thus lower disability of the patients. This contrasts our findings with the mRS and is, interestingly, also not consistent with the suggested loss of retinal nerve fibers as a sign of retinal neurodegeneration and a possible progression marker [23]. Of note, in that study, only for half of the patients was the ALS-FRS available and could be included in the follow-up analysis [23].

The small study population in our and other studies [18,22,23] might be a relevant factor for the inconsistent and in some cases contradictory results in the literature. Restrictive inclusion criteria and the rapid progressive course of the disease limit eligible participants. OCT measurement, for example, cannot be performed on patients who are not able to sit upright or in need of intensive care or ventilation. This might also influence the high rates of loss to follow-up and limited availability of longitudinal data in this and other studies. The short follow-up intervals in most studies also limit the power, as one might rather expect changes in OCT in a neurodegenerative disease after two to three years.

Rojas et al. and Rohani et al. used the CIRRUS® (Carl Zeiss Meditec AG, Jena, Germany), while Roth et al. [22] and we used the SPECTRALIS® device (Heidelberg Engineering GmbH). The evaluated grids differed in all studies, rendering comparisons difficult.

Heterogeneity of the involvement of different neurological systems might also play a role in the ophthalmological outcomes. A more detailed clinical differentiation and stratification of patients by symptoms in future analyses might be purposeful.

The above-mentioned small study cohort and short follow-up period are the main limitations of this study. We do, however, believe that despite these limitations, our data is still important and adds to the literature: Our study supports the notion of those studies reporting no differences in retinal layer thickness between patients with early ALS and HCs. The missing significant thickness changes in our longitudinal data suggest that longitudinal measurements may not be suitable for the monitoring of disease progression, especially over a limited period of time (up to 6 months). At the same time, longer monitoring intervals might be impractical due to loss to follow-up.

The role of OCT measurements in detecting disease progression in patients with ALS remains unclear, as we detected no relevant progression of retinal layer thinning. Longitudinal analysis over several years proves to be difficult because of the rapid progression and poor prognosis in some patients. With progress in genetic science and increased availability of genetic testing, new stratifications of cohorts and earlier diagnosis, resulting in longer follow-up times, may become possible. Thickness changes in pRNFL and mINL might prove themselves as a biomarker for early diagnosis or prodromic ALS. Data on monogenetic familial ALS might add more insight into the potency of OCT as a potential biomarker.

## Figures and Tables

**Table 1:** OCT results at baseline and annualized change rates for patients with ALS and healthy controls.

OCT Layer, µm ^a^	ALS	HCs	Statistics: ALS vs. HC
Baseline ^a^	(n = 22)	(n = 21)	GEE
pRNFL	93.20 (±1.83)	94.15 (±1.38)	*p* = 0.357
TMT	299.63 (±2.74)	300.18 (±2.01)	*p* = 0.711
mRNFL	32.44 (±0.67)	33.26 (±0.46)	*p* = 0.383
mGCIPL	65.74 (±1.02)	66.12 (±0.73)	*p* = 0.525
mINL	33.03 (±0.44)	32.87 (±0.31)	*p* = 0.773
mOPL	28.58 (±0.48)	28.19 (±0.28)	*p* = 0.399
mONL	60.46 (±1.33)	61.20 (±1.00)	*p* = 0.631
mRPE	13.90 (±0.18)	14.14 (±0.916)	*p* = 0.367
**Annualized Change Rate After 12 Months ^b,c^**			
	**(n = 22)**	**(n = 5)**	**Mann–Whitney ^d^**
pRNFL	−1.73 (±2.50); *p* = 0.928 ^b^ **(<0.001 ^c^)**	−0.18 (±1.93); *p* = **0.005 ^c^**	*p* = 0.864
TMT	0.94 (±1.85); *p* = 0.080 **(<0.001)**	0.70 (±0.22); *p* = **0.005**	*p* = 0.703
mRNFL	0.00 (±1.14); *p* = 0.071 **(<0.001)**	0.30 (±0.05); *p* = **0.005**	*p* = 0.182
mGCIPL	0.91 (±0.75); *p* = 0.402 **(<0.001)**	−0.31 (±0.05); *p* = **0.005**	*p* = 0.340
mINL	−0.48 (±0.71); *p* = 0.654 **(<0.001)**	−0.06 (±0.04); *p* = **0.005**	*p* = 0.849
mOPL	0.56 (±0.90); *p* = 0.274 **(<0.001)**	−0.12 (±0.02); *p* = **0.005**	*p* = 0.341
mONL	0.78 (±1.50); *p* = 0.867 **(<0.001)**	−0.09 (±0.04); *p* = **0.005**	*p* = 0.849
mRPE	0.15 (±0.45); *p* = 0.381 **(<0.001)**	−0.04 (±0.02); *p* = **0.005**	*p* = 0.503

Mean thickness at baseline and mean annualized thickness change after 12 months are presented for each retinal layer; standard error of the mean (SEM) can be found in parentheses. ^a^ Using GEE models correcting for inter-eye correlations and sex, baseline thickness of the retinal layers was compared between patients with ALS and healthy controls (HCs). ^b^ Changes after 12 months in comparison to baseline were analyzed using the same GEE model for patients with ALS. The results can be found after the semicolon behind the thickness change rate. ^c^ Thickness after 12 months was compared to baseline in HCs using the Wilcoxon test because of small sample size; for comparison, it was also analyzed for patients with ALS, and can be found in parentheses after the first *p*-value. ^d^ Annualized change rates between both cohorts were compared using the Mann–Whitney U test. pRNFL = peripapillary retinal nerve fiber layer; TMT = total macular thickness; mRNFL = macular retinal nerve fiber layer; GCIPL = complex of GCL = ganglion cell layer + IPL = inner plexiform layer; INL = inner nuclear layer; OPL = outer plexiform layer; ONL = outer nuclear layer; RPE = retinal pigment epithelium. The results were considered significant with *p* ≤ 0.05 and depicted in bold letters.

**Table 2:** Correlation between OCT parameters and clinical scores in patients with ALS.

OCT-Parameter, µm	mRS (n = 22)	BI (n = 9)	ALSFRS-R (n = 6)
Baseline			
pRNFL	0.15 (*p* = 0.969)	0.25 (*p* = 0.517)	−0.348 (*p* = 0.499)
TMT	−0.238 (*p* = 0.538)	−0.079 (*p* = 0.839)	−0.086 (*p* = 0.872)
mRNFL	−0.337 (*p* = 0.376)	0.129 (*p* = 0.741)	0.600 (*p* = 0.208)
mGCIPL	−0.396 (*p* = 0.291)	0.099 (*p* = 0.800)	−0.257 (*p* = 0.623)
mINL	−0.487 (*p* = 0.183)	0.03 (*p* = 0.939)	−0.464 (*p* = 0.354)
mOPL	−0.644 (*p* = 0.061)	0.119 (*p* = 0.761)	−0.314 (*p* = 0.544)
mONL	0.218 (*p* = 0.573)	−0.099 (*p* = 0.800)	−0.257 (*p* = 0.623)
mRPE	−0.219 (*p* = 0.572)	0.592 (*p* = 0.093)	0.314 (*p* = 0.544)
**Follow-Up**			
pRNFL	−0.375 (*p* = 0.286)	0.006 (*p* = 0.986)	0.347 (*p* = 0.399)
TMT	−0.391 (*p* = 0.338)	0.064 (*p* = 0.881)	0.406 (*p* = 0.425)
mRNFL	−0.587 (*p* = 0.126)	0.37 (*p* = 0.366)	**0.899 (*p* = 0.015)**
mGCIPL	**−0.761 (*p* = 0.028)**	0.54 (*p* = 0.168)	0.754 (*p* = 0.084)
mINL	−0.531 (*p* = 0.175)	0.373 (*p* = 0.363)	0.638 (*p* = 0.173)
mOPL	0.548 (*p* = 0.160)	−0.026 (*p* = 0.952)	−0.232 (*p* = 0.658)
mONL	−0.587 (*p* = 0.126)	−0.128 (*p* = 0.763)	0.174 (*p* = 0.742)
mRPE	0.156 (*p* = 0.711)	−0.549 (*p* = 0.159)	−0.464 (*p* = 0.354)

Using Spearman’s Rho, a possible correlation between the retinal layer thickness at baseline and the clinical scores, and between the annualized change rate after 12 months was analyzed. pRNFL = peripapillary retinal nerve fiber layer; TMT = total macular thickness; mRNFL = macular retinal nerve fiber layer; mGCIPL = complex of GCL = ganglion cell layer + IPL = inner plexiform layer; mINL = inner nuclear layer; mOPL = outer plexiform layer; mONL = outer nuclear layer; mRPE = retinal pigment epithelium. The results were considered significant with *p* ≤ 0.05 and printed in bold letters.

## Data Availability

By request of a qualified researcher, all data are available.

## Abbreviations

The following abbreviations are used in this manuscript:

TMT	total macular thickness
pRNFL	peripapillary retinal nerve fiber layer
mRNFL	macular retinal nerve fiber layer
GCIPL	complex of GCL = ganglion cell layer + IPL = inner plexiform layer
INL	inner nuclear layer
OPL	outer plexiform layer
ONL	outer nuclear layer
RPE	retinal pigment epithelium
ALS	amyotrophic lateral sclerosis
OCT	optic coherence tomography
HCs	healthy controls
ALS-FRS	ALS functional rating scale
mRS	modified Rankin scale
GEE	generalized estimation equation model
